# Application Progress of Multi-Functional Polymer Composite Nanofibers Based on Electrospinning: A Brief Review

**DOI:** 10.3390/polym16172459

**Published:** 2024-08-29

**Authors:** Shuai Ma, An Li, Ligang Pan

**Affiliations:** Institute of Quality Standard and Testing Technology, Beijing Academy of Agriculture and Forestry Sciences, Beijing 100097, China; lia@iqstt.cn (A.L.); panlg@iqstt.cn (L.P.)

**Keywords:** nanocomposite, polymer fiber, thin film, electrospinning

## Abstract

Nanomaterials are known as the most promising materials of the 21st century, among which nanofibers have become a hot research and development topic in academia and industry due to their high aspect ratio, high specific surface area, high molecular orientation, high crystallinity, excellent mechanical properties, and many other advantages. Electrospinning is the most important preparation method for nanofibers and their thin membranes due to its controllability, versatility, low cost, and simplicity. Adding nanofillers such as ceramics, metals, and carbon materials to the electrospinning polymer solutions to prepare composites can further improve the mechanical strength and multi-functionality of nanofibers and their thin membranes and also provide possibilities for their widespread applications. Based on the rapid development in the field of polymer composite nanofibers, this review focuses on polyurethane (PU)-based composite nanofibers as the main representative and reviews their latest practical applications in many fields such as sound-absorbing materials, biomedical materials (including tissue engineering implants, drug delivery systems, wound dressings and other anti-bacterial materials, health materials, etc.), wearable sensing devices and energy harvesters, adsorbent materials, electromagnetic shielding materials, and reinforcement materials. Finally, a summary of their performance–application relationship and prospects for further development are given. This review is expected to provide some practical experience and theoretical guidance for further developments in related fields.

## 1. Introduction

The size range of the structural components of nanomaterials is approximately 1–100 nm, and the small size effects, surface–interface effects, quantum effects, and tunneling effects generated by this extremely small size give them various properties that are completely different from macroscopic materials (i.e., bulk materials) [1]. Among them, as one-dimensional (1D) nanomaterials, nanofibers (usually referred to as polymer nanofibers) have characteristics such as a high aspect ratio, large specific surface area, high surface free energy, high molecular chain orientation, high crystallinity, and good mechanical properties (including high modulus, high toughness, and high tensile strength) [2]. Nanofibers, as outstanding representatives of nanomaterials, have a wide range of applications in filtration, insulation, noise reduction, and other fields. Their preparation methods include stretching into fibers, template synthesis, self-assembly, microphase separation, electrospinning, etc.

As the most efficient, large-scale, and low-cost method for preparing polymer nanofibers and their thin membranes, electrospinning utilizes electrostatic forces to produce polymer fibers with diameters ranging from nanometers to micrometers. In terms of history, electrospinning technology can be traced back to the study of Raleigh et al. in 1882 on the instability of droplets in an electric field and the study of Taylor et al. in 1915 on the fragmentation of droplets and charged fiber bundles in an electric field. The true electrospinning technology began with Formhals’ patent in 1934 for the process and apparatus of preparing polymer filaments using an electrostatic field [3]. Its large-scale applications began in the 1990s, advancing with the development of nanotechnology. Its specific modes mainly include needle (including single- and multi-needle) spinning, needleless spinning, and bubble spinning [4]. In the electrospinning process, the polymer solution needs to be prepared first and then placed in a strong electric field. At a specific field strength (i.e., critical field strength), the spherical droplets will be stretched into a cone (called a “Taylor cone”). When a charged jet is ejected from the cone, the cone will be extended into a straight line due to instability. At this point, the jet will be stretched into a finer straight line and solidified, depositing onto the collection plate and becoming nanofibers. Electrospinning can precisely control the diameter and morphology of nanofibers, as well as their membrane thickness, by changing the voltage, plate spacing, duration time, and other parameters. There are many types of polymers suitable for electrospinning, and the most commonly reported ones include cellulose acetate, polyacrylonitrile (PAN), polyamide (PA), polycaprolactone (PCL), polyketone (PK), polylactic acid (PLA), polymethyl methacrylate (PMMA), polystyrene (PS), polysulfone, polyurethane (PU), polyvinyl alcohol (PVA), polyvinylidene fluoride (PVDF), etc. [5]. Among them, PU, as an excellent thermoplastic polymer elastomer, has excellent comprehensive performances (such as light weight, wear resistance, tear resistance, pressure resistance, bending resistance, cold resistance, corrosion resistance, good thermal insulation, good biocompatibility, easy biodegradation, etc.) and good processibility (suitable for injection molding, extrusion, casting and so on) [6]. These properties make it highly popular in applications such as textiles, biomedical, anti-bacterial, sensing, and water filtration. The main disadvantages of PU are easy aging (susceptible to factors such as ultraviolet (UV) radiation, oxygen, temperature changes, etc.) and low mechanical strength and modulus, which need to be taken into consideration for practical applications. PU was invented by Otto Bayer in 1937 and can be divided into polyester type and polyether type, which can be used to prepare materials with different properties, such as plastics, fibers, and elastomers. Its chemical synthesis reaction is the condensation polymerization of polyisocyanate curing agents with diols, which are usually the rigid part and flexible part, respectively, in the system. The rigid part usually determines the hardness and high-temperature properties, while the flexible part determines the elasticity, low-temperature properties, and solvent resistance. Both the crosslinking density and crystallinity play important roles in the comprehensive properties. What makes PU an excellent electrospun nanofiber material is not only its wide range of applications and affordable price but also its good room-temperature solubility, multiple solvent options, suitable solution viscosity, and good flexibility.

**Figure 1:**
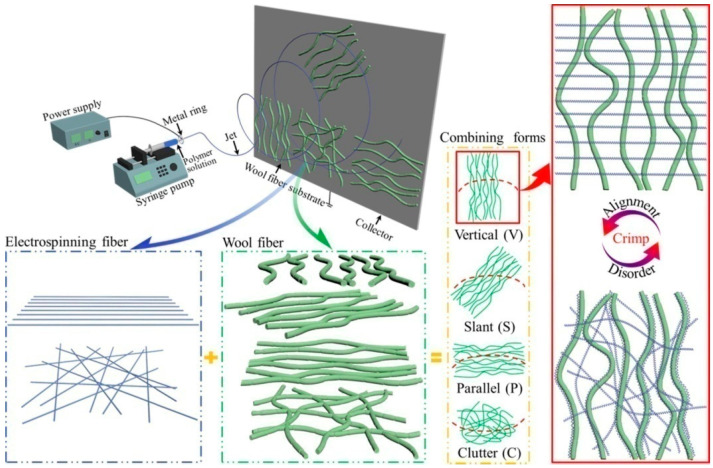
Schematic presentation of the overall preparation process of self-crimped electrospinning fiber deposited on wool fiber substrate, including spinning method, combining form and fiber morphology (reproduced with permission from [7]).

Compared with ordinary fiber cloth, electrospun nanofiber membranes have many advantages, such as large specific surface area, small pores, high porosity, light weight, ultra-low thickness, and flexible mechanical properties. Therefore, they have good application prospects in fields such as filtration, energy, catalysis, environmental protection, clothing, and tissue engineering. Electrospinning can also conveniently regulate the morphology of the resulting nanofibers. For example, curling is an important form of fiber structure that can improve the flexibility, elasticity, and spatial scalability of fibers. Xiong et al. proposed a strategy to form continuous self-crimped nanofibers through one-step electrospinning and found that the crimping of electrospun PS fibers deposited on a wool substrate was caused by the comprehensive differences in diameter, dielectric constant, conductivity, and gap width between the substrate and fibers (as shown in Figure 1) [7]. The corresponding theories were also experimentally verified. Such self-crimped nanofiber can be used in fields such as water conduction, insulation, and functional carriers.

Adding a small amount of nanoscale inorganic or metal fillers with a high specific surface area to the polymer matrix can provide additional functionality for the resulting composites, i.e., generate synergistic effects [8]. The methods for preparing nanocomposites mainly include the solution method, melting method, in situ growth method, etc. The nanomaterials that can be used as fillers include carbon nanomaterials (such as carbon nanotube (CNT), graphene, etc.), inorganic nanomaterials (such as clay, silica (SiO_2_), titanium dioxide (TiO_2_), etc.), metal or its oxide nanoparticles (such as gold (Au), silver (Ag), ferric oxide (Fe_3_O_4_), etc.), and quantum dots [9].

Combining polymer composite technology with electrospinning methods can not only significantly improve the mechanical and thermal properties of polymer nanofibers but also endow them with additional functions (such as light, electricity, magnetism, catalytic performance, etc.), thereby preparing more flexible functional polymer composite nanofibers [10]. Among them, the extremely large specific surface area of nanofibers is very conducive to their functionalization, thereby achieving improvement effects far superior to traditional fibers with typical diameters of a few micrometers.

**Figure 2:**
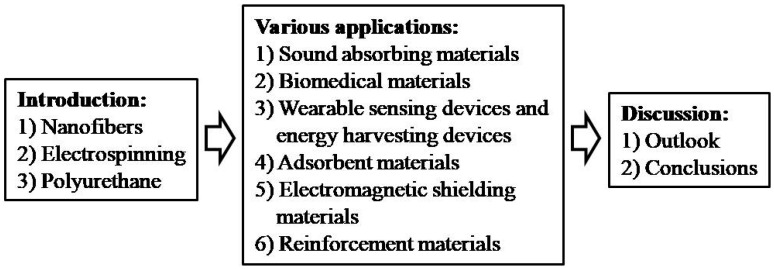
Schematic presentation of the main content of this review.

Based on the above background, this review intends to take the PU matrix as the main representative, focusing on overviewing the numerous practical applications of the polymer composite nanofibers and their thin membranes in fields of sound-absorbing materials, biomedical materials, wearable sensing devices and energy-harvesting devices, adsorbent materials, electromagnetic shielding materials, reinforcement materials, etc. (as shown in Figure 2). We will not only explore their latest progress but also strive to reveal the logical clues behind their development. This review can not only provide important practical experience for the further development of related fields but also provide useful theoretical and directional guidance for their future development direction.

## 2. Numerous Applications of Polymer Composite Nanofibers and Their Thin Membranes

### 2.1. Sound-Absorbing Materials

Noise pollution is the third largest environmental problem, only after water and air pollution, and is becoming increasingly serious with industrialization, urbanization, population growth, and the growth of roads, railways, and air transportation. Long-term exposure to noise pollution can lead to hearing impairment, sleep disorders, endocrine disorders, immune dysfunction, cardiovascular disease, and even cognitive impairment. The use of porous sound-absorbing materials prepared from fibers is an important and effective way to reduce noise pollution, and their sound absorption mechanisms can be divided into two types: porosity and resonance. Among them, the porous, sound-absorbing materials have a large number of internal pores. When sound waves enter the pores, they will rub against the air inside, thereby converting the sound energy into heat energy for consumption. In contrast, the resonant sound-absorbing materials consume the sound energy by resonating with sound waves through fibers and other components [11]. Traditional fiber sound-absorbing materials such as cotton, linen, and wool have good sound absorption ability for high-frequency noise above 2500 Hz, while the nanofiber thin membranes have better sound absorption ability for low-frequency noise below 500 Hz and the intermediate-frequency noise of 500–2500 Hz. Combining these two fibers may have the best overall sound absorption effect. Especially with the low thickness of nanofiber membranes, they can significantly enhance the performance of sound-absorbing materials with slight increases in material thickness and weight. In fact, the sound absorption mechanism of nanofiber membranes involves both porosity and resonance; therefore, their sound absorption effect is superior to that of traditional materials. Recently, Li et al. have reviewed the research progress of electrospun nanofiber sound-absorbing materials [12], pointing out that the diameter, morphology, surface morphology, spatial structure, and membrane thickness of nanofibers can be controlled by changing the main parameters during electrospinning (such as raw materials, processing parameters, and duration time) to regulate the sound-absorbing performance of nanofiber membranes.

**Figure 3:**
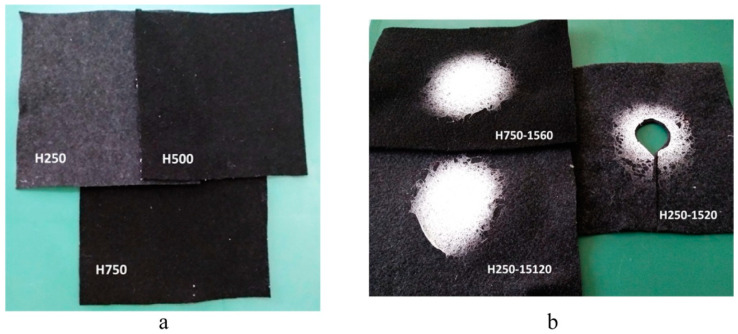
Photographs of the nonwoven PET fabrics made by recycled bottle waste (**a**) and the PU nanofiber membrane reinforced nonwoven fabrics (**b**) (reproduced with permission from [13]).

There is a lot of research in this area. For example, Ozkal et al. used a 15 wt% PU solution and continuous electrospinning for 5–120 min to prepare nanofiber membranes and then combined them with needle-punched nonwoven polyethylene terephthalate (PET) fabrics of different thicknesses with a weight of 250–750 g m^−2^ to prepare sandwich nanofiber-reinforced nonwoven fabrics (as shown in Figure 3) [13]. The results showed that the material had good sound absorption performance, especially in the low frequency range, which could achieve good noise control. It was also found that as the thickness of the nanofiber membrane increased, the resonance frequency of the material decreased, and the sound absorption effect was improved. It is worth noting that the needle-punched nonwoven PET fabric they used was prepared from recycled plastic bottle waste, which was extremely important for environmental protection. In fact, PET is one of the most common plastic bottle wastes, with good mechanical and processing properties. After recycling, it can be used to prepare textiles such as nonwoven fabrics. Selvaraj et al. also adopted the same sustainable economic approach, which involved recycling useful substances from leather industry waste for use as sound-absorbing materials [14]. Specifically, the collagen hydrolysate extracted from waste leather scraps was electrospun together with PVA and then sandwiched between PAN nanofiber layers to prepare multi-layer composite sound-absorbing materials. It was found that the multi-layer composites exhibited good sound absorption performance in the frequency range of 800–2500 Hz, and their thermal stability and tensile strength were better than those of PAN nanofiber membranes. Similarly, Wu et al. used recycled PET plastic bottles, oyster shell powder, and SiO_2_ aerogel to prepare the composite textiles [15]. The PET in that product had high hydrophilicity and low thermal conductivity but no anti-bacterial effect. The addition of oyster shell powder could improve its anti-bacterial performance, and the addition of SiO_2_ aerogel could enhance the hydrophobicity, reduce the thermal conductivity, and thus achieve better temperature control. Textiles, as the interface between the human body and the environment, are crucial for human health and comfort. The ternary composite textile became a functional material with good application prospects due to its excellent tensile strength, insulation, thermal stability, temperature controllability, water resistance, washing resistance, and anti-bacterial properties.

**Figure 4:**
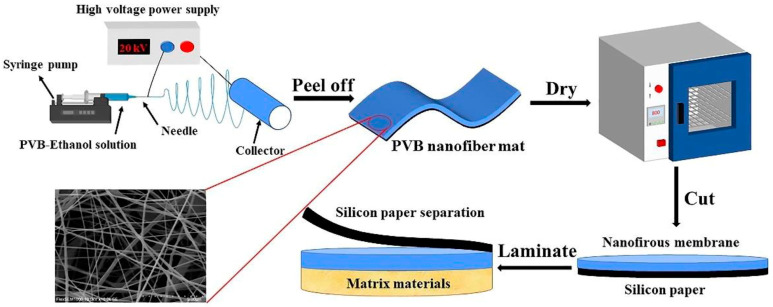
Schematic presentation of the preparation process of the multi-layer composite nanofiber membrane [16]).

Similarly, in response to the poor sound absorption performance of traditional fiber sound-absorbing materials in the low frequency range, Shao et al. prepared the polyvinyl butyral (PVB)/PET bilayer and PVB/PET/thermoplastic polyurethane (TPU) three-layer composite sound-absorbing materials by laminating PVB nanofiber membranes, PET fiber felt, and TPU membranes (as shown in Figure 4) [16]. They found that when the nanofiber membrane was placed on the sound-absorbing surface, its sound absorption performance was better in the low-frequency range of 100–2500 Hz. By selecting and optimizing the structural parameters reasonably, the sound absorption performance of different frequency bands could be improved in a targeted manner. For example, as the thickness of the PVB nanofiber membrane increased, its resonance sound absorption peak shifted toward the low frequency range. The comparison showed that the three-layer structure had superior sound absorption performance in the low-frequency range compared to the double-layer composite structure material. The reason why the addition of PVB nanofiber membrane could significantly improve the sound absorption performance of PET fiber felt was due to the coupling of porous structure and resonance sound absorption mechanism.

**Figure 5:**
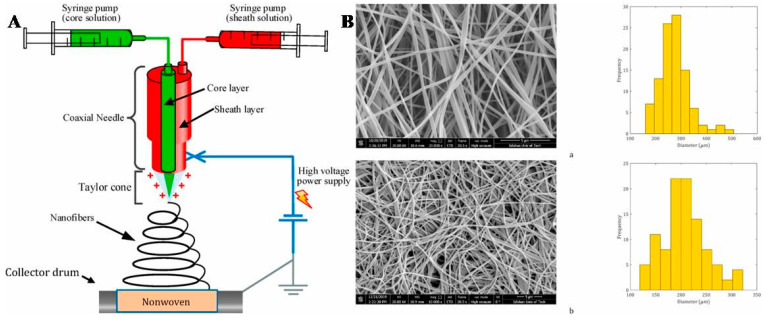
Schematic presentation of the co-axial electrospinning setup (**A**) and scanning electron microscope (SEM) images and nanofiber diameter distributions of core–shell (**a**) and hollow (**b**) nanofibers (**B**) (reproduced with permission from [17]).

Considering that most fiber absorbers currently used in buildings cannot fully meet the requirements for low-frequency sound absorption, Hajimohammadi et al. combined the nanofiber structures with traditional fiber absorbers to achieve better results without increasing the weight and thickness of the absorbers [17]. Their specific method was to add a solution of polyvinyl acetate (PVAc) and PAN to a co-axial spinneret to prepare the core–shell structured nanofibers and then immerse the core–shell structured nanofibers in hot water to extract the PVAc cores, finally obtaining the PAN hollow nanofibers (as shown in Figure 5). They placed these two types of electrospun nanofiber membranes on the front or back of nonwoven fabrics to prepare the composite fiber membranes. The results indicated that compared with the core–shell structured nanofibers, the hollow-structured nanofibers had superior sound absorption performance. Their research indicated that when the nanofiber membranes were electrospun onto the back of nonwoven fabrics, their sound absorption mechanism belonged to the anti-adhesive type, and their sound absorption performance increased with increasing nanofiber membrane thickness. When the nanofiber membrane was electrospun onto the front of the nonwoven fabric, its sound absorption mechanism was resonance. The sample exhibited good sound absorption performance in the low- and mid-frequency bands, and as the film thickness increased, its absorption peak shifted toward lower frequencies, and the sound absorption efficiency was also improved.

Similarly, to determine the effect of nanofiber membranes placed on rigid and flexible substrates on the sound absorption performance, Karaca et al. investigated the sound absorption performance of TPU and TPU/PS nanofiber membranes combined with rigid glass fiber fabric-reinforced epoxy composites and flexible polypropylene (PP) spun-bond nonwoven fabric [18]. They also studied the TPU nanofiber membranes and TPU/PS fiber membranes before and after the Soxhlet extraction. The results showed that the impact mechanisms of elastic nanofiber membranes on the sound absorption performance of rigid glass fiber fabric epoxy composites and flexible/soft nonwoven fabrics were completely different. The number of fibers in a nanofiber membrane that was effective in friction damping was more effective in promoting the sound absorption performance of rigid glass fiber fabric epoxy composites, while the elastic vibration damping of nanofiber membranes was more effective in absorbing the sound of flexible/flexible nonwoven materials. When the nanofiber membrane was combined with nonwoven fabric, the TPU/PS composite nanofiber membrane after the Soxhlet extraction had better sound absorption performance than the TPU fiber membrane because of its higher ductility. When the nanofiber membrane was integrated with a rigid glass fiber fabric epoxy resin composite layer, the TPU nanofiber membrane could provide a better sound absorption effect. That was because the diameter of the PU nanofiber membrane was smaller, and the number of fibers in it was also higher, resulting in higher friction damping. Karaca et al. further compared the sound absorption performance and thermal conductivity of the TPU/PS composite nanofiber network, TPU nanofiber network, and TPU/PS microfiber network after the Soxhlet extraction [19]. The TPU/PS microfiber network after the Soxhlet extraction showed the best absorption performance at low frequencies (600–800 Hz), the TPU nanofiber network was more effective at high frequencies (about 3000 Hz), and the TPU/PS composite nanofiber network had the best effect at medium frequencies (about 1000 Hz). In addition, the thermal conductivity of the TPU/PS composite nanofiber membrane was the lowest (about 0.05 W m^−1^ K^−1^).

In terms of theory, Sakamoto et al. used four estimation models, including the Rayleigh model, the Miki model, the Komatsu model, and the Simplified Limp Frame model, to calculate and simulate the sound absorption principle of nanofiber membranes, especially the problem of transmission loss, and compared them with experimental data [20]. It was found that the Simplified Limp Frame model was closer to the actual experimental results than traditional models such as the Rayleigh, Miki, and Komatsu models.

### 2.2. Biomedical Materials

The applications of electrospun nanofibers in the biomedical field mainly include implantable materials (such as scaffolds) in tissue engineering, controlled drug release, wound dressings, and health materials.

#### 2.2.1. Implantable Materials for Tissue Engineering

Tissue engineering is an emerging discipline that combines cell biology and material science to construct tissues or organs in vitro or in vivo [21]. Tissue engineering is crucial for biomimetic grafts such as artificial bones, blood vessels, and hearts to replace autologous tissue. For example, natural bone is usually composed of collagen scaffolds and uniaxially oriented apatite crystals, which can be regarded as a natural composite reinforced by hydroxyapatite. One of the most critical issues in bone tissue engineering is to design biomaterials with similar mechanical properties to natural bone to affect bone structural development and promote osteogenic differentiation of bone mesenchymal stromal cells. In addition, autologous vascular transplantation is limited in cardiovascular surgery as it typically requires invasive surgery at the donor site and may lead to potential complications, requiring tissue engineering to prepare artificial blood vessels. For large-diameter blood vessels, synthetic grafts such as polyester and expanded polytetrafluoroethylene (PTFE) are effective alternatives. However, for small-diameter (less than 6 mm) blood vessels, it is necessary to rely on three-dimensional (3D) bioprinting or electrospinning techniques in tissue engineering. Similarly, myocardial infarction is one of the most common cardiovascular diseases, which can lead to widespread cell death, loss of anisotropic cellular structure, and remodeling of heart tissue. One potential solution for restoring the function of infarcted heart tissue is to reconstruct the in vitro heart tissue structure through tissue engineering and introduce it into damaged heart tissue in the body.

**Figure 6:**
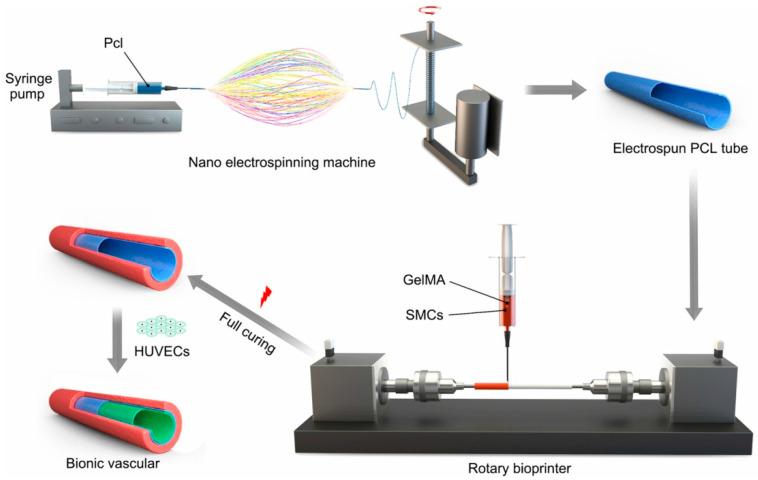
Schematic presentation of the fabrication of a small-diameter bionic vascular tissue by combining nanofiber electrospinning and rotary bioprinting (reproduced with permission from [23]).

There is a lot of research and development in this area. For example, considering that the anisotropic structure of the heart tissue in vivo was crucial for the effective pumping of blood in the native heart, Eom et al. achieved the preparation of multi-layer scaffolds with different orientations for each layer in the development of the heart tissue structure in vitro [22]. The basic idea was to prepare the nanofiber pads with a thickness of less than 10 μm by electrospinning. Not only could it generate uniaxial contraction motion, but it also had mechanical stability. Jin et al. combined the nanofiber electrospinning with a rotating bioprinter to create a biomimetic small-diameter artificial blood vessel graft containing smooth muscle cells and endothelial cells (as shown in Figure 6) [23]. Among them, electrospun PCL had good elasticity, which was beneficial for the adhesion and functionalization of endothelial cells. The rotating bioprinter arranged cells along a horizontal axis of rotation. Therefore, the biomimetic structure was superior to natural blood vessels in terms of elasticity, anti-burst pressure, and suture retention strength and was expected to be applied in the biomedical fields such as urethra, trachea, and intestine.

To develop bone repair materials with excellent mechanical properties and stem cell affinity, Di et al. prepared a 3D PLA acid-based composite nanofiber membrane that was compatible with bone marrow stem cells by grafting poly(*D*-lactic acid) (PDLA) onto hydroxyapatite and enantiomeric PLA [24]. Compared with the poly(*L*-lactic acid) (PLLA) nanofiber membrane, the tensile strength and Young’s modulus of the composite nanofiber membrane were increased by 30.2% and 34.6%, respectively, mainly due to the formation of 3D composite microcrystals of PLA. In addition, it had good heat resistance and biocompatibility.

#### 2.2.2. Drug Delivery System

To avoid healthy cells and only target the cells affected by cancer in the body, a targeted drug delivery system is needed [25]. With the emergence and development of nanotechnology, many nanoparticles have been found to be particularly suitable for targeted drug delivery systems and have become an effective method for improving cancer treatment. The polymer (natural, synthetic, or both) nanofibers prepared by electrospinning have multiple advantages over nanoparticles when used in targeted drug delivery systems. Meanwhile, when used for targeted drug delivery, the polymer is usually biodegradable to avoid its residue and accumulation in the human body. Recently, Aminu et al. reviewed the application progress of electrospun nanofiber targeted drug delivery in cancer treatment in response to this research hotspot [26]. They pointed out that the reason why electrospun nanofibers could be used as good drug nanocarriers was due to their outstanding advantages such as high specific surface area, high porosity, excellent controllability, high encapsulation efficiency, high biodegradability, high biocompatibility, and low cost.

Rad et al. investigated the synergistic anti-cancer effect of PU-PCL copolymer nanofibers on the prostate cancer cell lines and evaluated their in vitro anti-tumor activity for the drug delivery system by using electrospun nanofibers for controlling the delivery of anti-cancer drugs and local chemotherapy of cancer [27]. The results indicated that the nanofiber had a significant synergistic therapeutic effect on the prostate cancer cells. They also pointed out that to achieve the ideal state of controlled drug release, the drug release rate could be controlled by changing the hydrophilicity of the polymer.

Ercelik et al. developed a nanofiber-based approach for local treatment of recurrent glioblastoma, addressing the issue of postoperative recurrence [28]. Specifically, the core–shell structured nanofibers were prepared by encapsulating PLA with Oleuropein, rutin, and temozolomide using the electrospinning technique. The nanofiber network could be used for controlled drug release, thus having a good therapeutic effect on the recurrence of glioblastoma.

#### 2.2.3. Wound Dressings and Other Anti-Bacterial Materials

Wound healing is a complex biological process, and wound dressings can act as a protective barrier against infection and promote wound healing [29]. To prevent bacterial colonization and subsequent wound infection, the surface of the wound dressing should be able to resist or prevent bacterial attachment, colonization, and proliferation. Traditional dressings such as gauze, silk, and bandages can generally only prevent bacterial infections, while new bioactive wound dressings can also help with wound healing. Among them, electrospinning nanofiber membranes are an important choice.

Hosseini et al. prepared the PVA nanofibers (about 203 ± 51 nm in diameter) containing chitosan nanoparticles and drug-loaded graphene oxide (GO)–Fe_3_O_4_ by electrospinning and used them as intelligent wound dressings for burns and diabetes wounds [30]. Specifically, the chitosan nanoparticles were synthesized by the ionic gel method, the GO–Fe_3_O_4_ nanocomposites were synthesized by the coprecipitation method in a nitrogen atmosphere, and then drugs were loaded on the nanoparticles and GO–Fe_3_O_4_ nanocomposites, and finally, nanofibers were prepared by electrospinning using PVA as raw material. The nanofiber membrane had a high water absorption capacity; therefore, it had a high absorption capacity for wound secretions. It also had low and suitable biodegradability, meeting the requirements of wound dressings. The presence of chitosan nanoparticles can produce a continuous and slow release, the presence of GO can prevent sudden drug release, and the presence of Fe_3_O_4_ can stimulate the intelligence of wounds. The experiments showed that the nanofiber membrane had good anti-bacterial properties against both Staphylococcus aureus (Gram-positive bacteria) and Escherichia coli (Gram-negative bacteria) and had high compatibility with cells.

**Figure 7:**
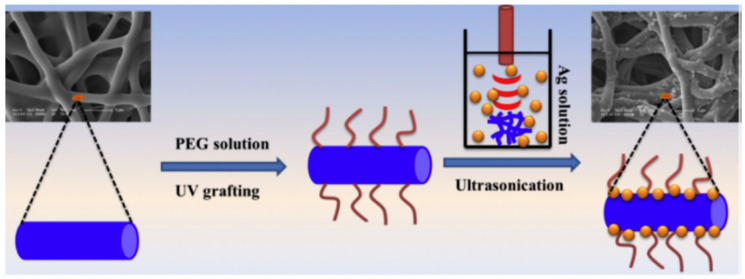
Schematic presentation of the preparation procedure of TPU-*g*-PEG/Ag composite nanofiber (reproduced with permission from [31]).

Shi et al. also developed similar anti-infective wound dressings [31]. Specifically, Ag nanoparticles were anchored onto the surface of TPU grafted polyethylene glycol (PEG) (TPU-*g*-PEG) nanofibers using ultrasound assistance (as shown in Figure 7). Among them, PEG was grafted onto TPU nanofibers by UV light, and then Ag nanoparticles were fixed on the surface of TPU-*g*-PEG nanofibers with ultrasound assistance. The TPU-*g*-PEG/Ag composite nanofiber had excellent blood compatibility, including the ability to inhibit platelet and red blood cell adhesion, lower hemolysis rate, and higher coagulation index. Moreover, due to the anti-bacterial effect of PEG and the bactericidal effect of Ag nanoparticles, the composite nanofiber exhibited good anti-bacterial performance in the in vitro testing using both Gram-negative and -positive bacterial strains.

Parin et al. prepared the TPU/PCL composite nanofibers loaded with spirulina biomass using the electrospinning method. The nanofibers had superhydrophilicity and high anti-bacterial activity against Escherichia coli, making them suitable for wound dressings and tissue engineering [32]. The average diameter of the nanofibers in the composites was about 228–312 nm, and the porosity was as high as 86–90 vol%. The composite nanofiber membrane significantly improved its swelling ability due to the addition of spirulina, and its tensile strength and Young’s modulus were also improved.

In addition to wound dressings, developing other types of anti-bacterial products with therapeutic properties is also very important for human health and has become a research and development hotspot. For example, in order to research and develop multi-functional textiles, Lee stacked electrospun PU/zinc oxide (ZnO) composite nanofiber membranes on cotton substrates to endow them with UV protection and anti-bacterial functions [33]. It was found that when the content of ZnO nanoparticles was 1 wt%, and the nanofiber membrane area density was 3.7 g m^−2^, the transmission of UV radiation could be significantly reduced. The composite nanofiber membrane/cotton fabric containing 5 wt% ZnO nanoparticles had an inhibition rate of over 98% against Staphylococcus aureus and Klebsiella pneumoniae. After adding 0.5 wt% Ag nanoparticles, the anti-bacterial performance of the composite fiber membrane could be further improved.

**Figure 8:**
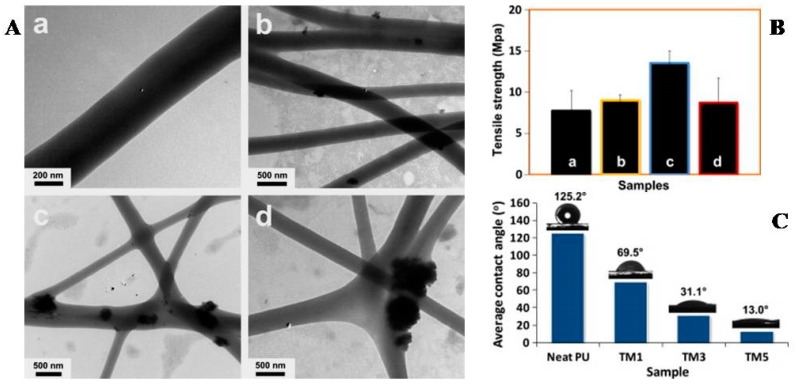
TEM images (**A**), tensile strength (**B**), and average contact angles (**C**) of electrospun nanofibrous mats of neat PU and various composites: neat PU (**a**), TM1 (**b**), TM3 (**c**), and TM5 (**d**) (reproduced with permission from [34]).

Tijing et al. prepared PU composite nanofibers filled with tourmaline (TM) nanoparticles (TN) with superhydrophilic and anti-bacterial properties through a one-step electrospinning process (as shown in Figure 8, where TM1, TM3, and TM5 are the composites with 1 wt%, 3 wt%, and 5 wt% TM nanoparticles, respectively) [34]. Due to the presence of a large amount of hydrogen bonding between the filler and the matrix, the dispersion state of the filler was good. It was found that adding 3 wt% filler could increase the tensile strength and elastic modulus of the material by 75% and 87%, respectively. In addition, the addition of fillers also improved the hydrophilicity of the material. When the filler content was 5 wt%, its water contact angle could be as low as 13°, reaching a superhydrophilic level. The addition of fillers significantly enhanced the regional inhibitory effect of the material on Escherichia coli (Gram-negative) and Enterococcus (Gram-positive), and the inhibitory effect also increased with increasing filler content. These excellent properties provided assurance for their use as anti-bacterial materials in the fields of hygiene protection textiles and water filtration.

#### 2.2.4. Health Materials

Recently, the popularity of mineral-based wearable therapies has flourished. Among them, negative ions are the most famous natural mineral source with therapeutic effects, usually referring to small ions with additional electrons. They are produced by various effects of thunderstorms, waves, evaporation, and minerals on water and oxygen in the air and exist at different concentrations in different environments [35]. They are also known as “air vitamins” and have functions such as anti-bacterial, bactericidal, and health benefits. They have a very important impact on the life activities of humans and other organisms. Their therapeutic ability is to neutralize free radicals in the body, thereby helping to purify the blood, promote blood circulation, stimulate cell metabolism, and reduce cell damage. Tourmaline is a material that can release negative ions. It is a crystalline borosilicate mineral that combines with elements such as aluminum (Al), iron (Fe), magnesium (Mg), sodium (Na), lithium (Li), or potassium (K). Tourmaline exhibits piezoelectric and pyroelectric properties under changes in temperature or pressure conditions, thus forming potential differences and exhibiting spontaneous surface electric fields. With this special property, tourmaline crystals can ionize surrounding water and oxygen molecules, spontaneously and permanently producing negative ions. Due to its negative air-release function, tourmaline is widely used in healthcare products and air or water purification. More importantly, people have always hoped that tourmaline particles could be well-loaded into polymer fibers, thereby further expanding the functionality and health of wearable products.

**Figure 9:**
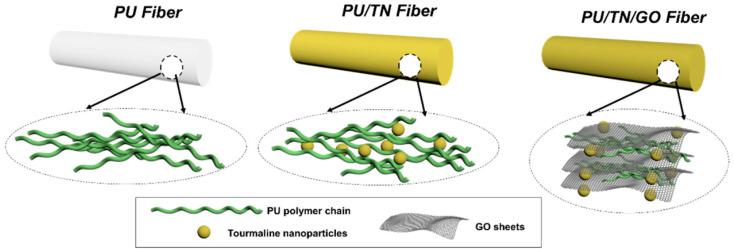
Schematic presentation of the PU and its TN and GO composite nanofibers (reproduced with permission from [36]).

In order to use energy therapy, including TN/negative ions, to improve human health, Zhang et al. addressed the shortcomings of TN-filled PU composite nanofibers in terms of mechanical properties and poor negative ion release performance. They chose GO as the modified filler and then used the wet spinning to prepare the composite nanofibers (as shown in Figure 9) [36]. The results showed that compared with unmodified composite nanofibers, Young’s modulus was increased by about 200%, the tensile strain at break was also significantly increased, and the number of negative ions was increased by 17 times and 1.6 times, respectively, compared to pure PU nanofibers and PU/TN nanofibers. The material was particularly suitable for applications in wearable energy therapy products.

### 2.3. Wearable Sensing Devices and Energy-Harvesting Devices

#### 2.3.1. Wearable Sensing Devices

Wearable sensing devices have been widely used in fields such as flexible electronics, electronic skin, and health diagnosis [37]. For example, exercise is an important component of a healthy lifestyle, and smart wearable devices can not only improve the quality of exercise but also provide health monitoring. For example, flexible pressure sensors can convert external stimuli into electrical pulses to track the biomedical parameters of the human body, thus having great potential in disease monitoring and diagnosis. Compared with the traditional strain sensors based on metal foils and semiconductors, wearable strain sensors based on conductive polymer composites have the advantages of low flexibility, small working range (<5%), light weight, high flexibility, and good processability. Unlike traditional electronic products based on rigid semiconductor chips and circuit boards, flexible electronic products used for wearable devices can withstand deformation such as tension, compression, bending, and twisting while also maintaining a conductive path under large strains. In addition, it also needs to have advantages such as light weight, low power consumption, high sensitivity, good corrosion resistance, high durability, high reliability, good skin friendliness, and good breathability. The conductive electrospun nanofibers can serve as key components of flexible electronic devices due to their excellent mechanical properties, high specific surface area, graded porous structure, and surface and component adaptability. Recently, Mercante et al. reviewed the application progress of the conductive electrospun nanofiber materials in the field of flexible electronics [38].

The basic idea of strain sensors based on conductive polymer composites is to introduce conductive nanofillers, such as graphene, CNT, metal and metal oxide nanowires, metal–organic frameworks (MOFs), two-dimensional transition metal carbon nitrides (MXenes), and conductive polymer nanofibers, into the synthetic or natural polymer matrices to create conductive networks. The strain sensing mechanism usually occurs when it is subjected to external force and produces strain, causing the resistance to increase. Then, it can return to its initial value after the strain is released. For example, Bhattacharyya et al. incorporated conductive nanofillers (including metal-coated nano graphite and carbon nanofibers) into TPU and then prepared composite nanofibers through a melt composite route using a micro twin-screw extruder and fiber stretching device [39]. The optimized processing parameters included processing temperature, mixing time, and screw speed. It was found that the metal-coated graphite nanoparticles improved the thermal stability of composite nanofibers. In addition, due to the formation of conductive networks, an extremely low amount of nanofillers could significantly reduce the direct current (DC) conductivity and alternating current (AC) impedance of the composite nanofibers. Therefore, such material can be used as an anti-static tow in fields such as biomedical and national defense.

**Figure 10:**
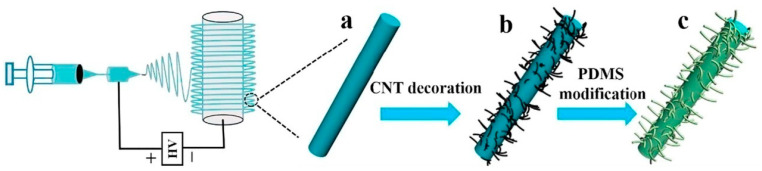
Schematic presentation of the preparation of TPU/CNT/PDMS composite nanofibers: TPU nanofiber (**a**), TPU/CNT composite nanofiber (**b**), and TPU/CNT/PDMS composite nanofiber (**c**) (reproduced with permission from [40]).

Wang et al. attached CNTs to the surface of electrospun TPU nanofibers by using ultrasound and then modified them with polydimethyl siloxane (PDMS) to prepare a superhydrophobic TPU/CNT/PDMS strain sensor (as shown in Figure 10) [40]. In terms of its working mechanism, CNTs are uniformly dispersed and anchored on the surface of TPU nanofibers intertwined with each other, forming a conductive network. The PDMS layer with low surface energy endowed the material with superhydrophobicity and anti-corrosion properties. The results showed that the introduction of CNT/PDMS simultaneously improved Young’s modulus, tensile strength, elongation at break, and the repetitive stability of the TPU nanofibers. Such a strain sensor could be used to detect various body movements (such as finger bending, elbow movements, squatting, and nodding) even in harsh environmental conditions such as humidity, acidity, and alkalinity.

Li et al. first used graphene to modify electrospun TPU nanofibers through ultrasound, then modified them with polydopamine (PDA), and finally hydrophobically treated them with 1H,1H,2H,2H-perfluorododecanethiol (PFDT) [41]. The mechanical properties (including Young’s modulus, tensile strength, and elongation at break) of the obtained conductive polymer composite nanofibers were significantly improved compared to the TPU nanofiber membrane. When used in strain sensors, it exhibited high tensile strength, controllable sensitivity, excellent cyclic stability, and durability. Such a nanofiber composite strain sensor could be attached to the skin for precise monitoring of different human movements (including subtle and large body movements), thus having great application prospects in wearable devices.

**Figure 11:**
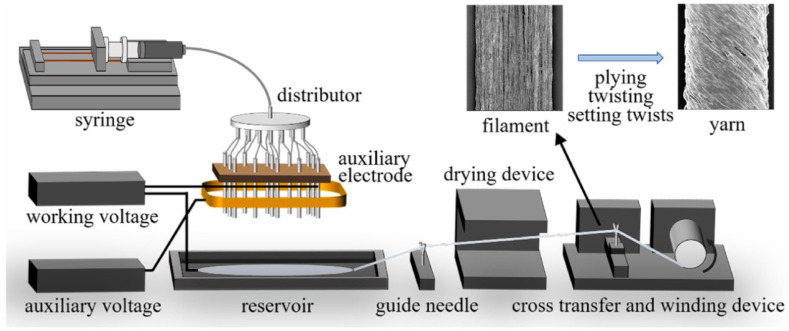
Schematic presentation of a device for electrospun nanofiber yarn (reproduced with permission from [42]).

Intelligent textiles with motion detection functions are very important for human health. To prepare conductive composite yarns with high strain sensing performance that can be directly integrated into fabrics through textile technology, Tang et al. uniformly dispersed CNT into a flexible TPU matrix and performed the multi-needle liquid bath electrospinning (as shown in Figure 11) [42]. The fracture elongation of the CNT/TPU composite nanofiber yarn was as high as 476%. In addition, they prepared the high conductivity impregnation coated CNT/TPU composite nanofiber yarns (with a resistivity of approximately 1.02 kΩ cm^−1^) by simply impregnating the CNT ink. The strain sensor based on this yarn exhibited a relative resistance change of up to 440% at a strain of 140% and had good linearity and repeatability. Finally, by directly sewing the composite yarn into an elastic self-adhesive bandage, an intelligent sports bandage with motion assistance and medical monitoring functions for heartbeat and respiration could be prepared.

#### 2.3.2. Energy Harvester

Most strain sensors are powered by backup batteries, which makes the device too bulky. In addition, the batteries used must be frequently charged or replaced, which obviously cannot meet the needs of wearable electronic products. The energy collection technology that converts the mechanical energy, solar energy, thermal energy, and other energy in the surrounding environment into charges can provide power for wearable electronic products and meet the growing demand for renewable energy and environmental protection requirements. In fact, the low-frequency mechanical energy (body movements) generated by the human body can be converted into electrical energy in wearable devices, which requires highly efficient energy-harvesting devices [43]. An example of this technology is the nanogenerator, which not only converts mechanical energy into electrical energy but also has many advantages, such as compact structure, stable performance, and low price, making it particularly suitable for use in self-powered equipment. Static induction and contact electrification are the main operating mechanisms of nanogenerators.

Among various energy-harvesting devices, flexible, wearable energy-harvesting devices have attracted widespread research attention due to their ability to improve energy-harvesting efficiency based on human motion while ensuring flexibility. Among them, due to the inherent flexibility of polymers, polymer-based energy harvesters can be used in many portable and lightweight applications, solving the problem of insufficient flexibility related to piezoelectric ceramics. These energy harvesters provide the possibility of controlling electronic devices at any time, making them widely applicable in areas such as health monitors, soft robots, flexible solar cells, electric skins, and flexible energy harvesters.

Gunasekhar et al. prepared the PVDF-based triboelectric nanogenerators (TENGs) through electrospinning to fabricate mechanical energy harvesters for wearable and portable electronic devices [44]. The specific process of preparing this energy harvester was to add the third-generation aromatic hyperbranched polyester as a filler to PVDF and then prepare the nanofibers through electrospinning. They found that the addition of fillers could increase the open circuit voltage of TENG by about eight times and the short circuit current by about 1.6 times.

Ni et al. prepared a flexible energy harvester based on the lead zirconate titanate (PZT)/shape memory PU nanofibers and found that the oriented arrangement of energy harvesters had better piezoelectric performance compared to the randomly arranged nanofibers [45]. It could effectively convert mechanical energy into electrical energy when subjected to different body movements such as bending, twisting, and applying pressure. Such an energy harvester had wearable characteristics.

### 2.4. Adsorbent Materials

The increasing demand for oil due to rapid industrialization has led to an increase in oil exploration and transportation through the ocean, and the accidents and spills have exacerbated the oil pollution, seriously affecting the aquatic organisms and the marine environment. This requires the development of cost-effective oil adsorbents. Among them, the magnetic adsorbent nanomaterials can recover the adsorbent of adsorbed oil from water through a magnetic field, thus avoiding secondary pollution. For example, Anushree et al. prepared the superparamagnetic PVDF composite nanofiber membranes using electrospinning technology and studied their adsorption performance for removing the oil sludge [46]. The water contact angle of the prepared fibers was 119–128°. Moreover, the addition of magnetic nanoparticles of manganese oxide (Mn_3_O_4_) increased the conductivity in polymer solutions, resulting in a decrease in the fiber diameter from 164 nm to 78 nm.

Hou et al. prepared an electrospun nanofiber membrane of PK and applied it to the separation of oil/water lotion [47]. They controlled the fiber diameter and pore size of the membrane by introducing sodium hydroxide (NaOH) into the precursor solution before the electrospinning and controlled the surface properties of the nanofibers by removing NaOH with water and reducing the functional groups with sodium borohydride (NaBH_4_), achieving superhydrophilicity between air and water and superhydrophobicity underwater. The results showed that the separation efficiency and recoverability of the fiber membrane for various oil/water lotions were higher than 97%.

**Figure 12:**
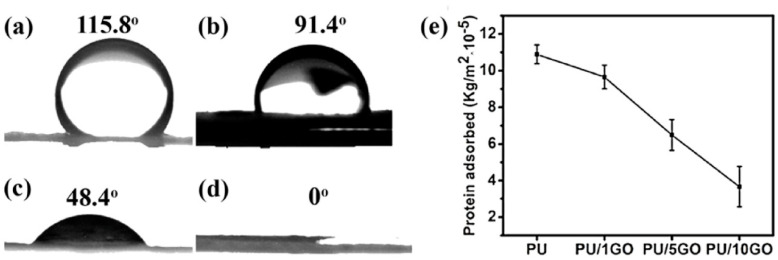
Photographs of water droplets showing contact angles of PU (**a**) PU/1 wt% GO (**b**), PU/5 wt% GO (**c**), and PU/10 wt% GO (**d**) membranes, and amount of protein adsorbed on different PU/GO membranes (**e**) (reproduced with permission from [48]).

Sundaran et al. prepared an electrospun PU/GO membrane for adsorbing organic dyes such as methylene blue and rhodamine B (as shown in Figure 12) [48]. The water flux of the super hydrophilic membrane was up to 17,706 L m^−2^ h^−1^; the maximum adsorption capacity for methylene blue and rhodamine B was up to 109.9 mg g^−1^ and 77.2 mg g^−1^, respectively; and the separation efficiency for oil in water lotion was up to 99.99%. The film also had both anti-fouling and anti-bacterial properties and had anti-bacterial activity against both Gram-negative and Gram-positive bacteria. The hydrogen bonding interaction between PU and GO in the composite nanofiber membrane gave it good thermal and mechanical stability. These properties have given the composite nanofiber membrane good prospects for application in water treatment.

### 2.5. Electromagnetic Shielding Materials

With the rapid development of electronic and wireless technology, electromagnetic radiation pollution is becoming increasingly serious. It not only causes interference with electronic devices but also seriously threatens human health. Therefore, there is an urgent need to develop high-performance electromagnetic shielding materials to reduce electromagnetic radiation pollution [49]. Traditional metal materials mainly achieve electromagnetic shielding through reflection, but their high density and susceptibility to corrosion limit their application in different environments. The conductive polymer composites are lightweight, low-cost, and easy to process, making them excellent electromagnetic shielding materials and excellent substitutes for metal materials.

Inspired by the hierarchical structure of the pearl layer, Li et al. prepared a lightweight, flexible, and superhydrophobic SiO_2^−^_ and Ag-coated PAN composite nanofiber membrane with high-performance electromagnetic interference shielding through electrospinning [50]. They first incorporated SiO_2_ into the PAN electrospun film and then deposited Ag nanoparticles with SiO_2_ as the adhesion site to obtain the core–shell structured nanofibers. The composite nanofiber membrane had superhydrophobicity (water contact angle reached about 157.0°), high conductivity (about 1.8 × 10^4^ S m^−1^), high specific shielding efficiency (367 dB cm^3^ g^−1^), and excellent conductivity stability in both acidic and alkaline solutions.

Huang et al. prepared the PTFE/multi-wall carbon nanotubes (MWCNTs) nanofiber membrane by combining the lotion electrospinning and high-temperature sintering [51]. It was found that compared with pure PTFE nanofiber membranes, a very low amount of CNT addition could not only significantly improve its mechanical properties (0.5 wt% CNT addition could increase the tensile strength of composite nanofiber membranes from 2.5 MPa to 5.9 MPa and the elongation at break from 123% to 330%) but also significantly improve its conductivity (5 wt% CNT addition could make the conductivity of nanofiber membranes reach 1.5 S m^−1^). The material also had good hydrophobicity, lipophilicity, and breathability. It could not only be used for electromagnetic shielding but also for electronic packaging, electrical engineering, and oil–water separation. It is worth noting that although PTFE membranes are widely used as filtration and distillation materials in fields such as textiles, electronics, electrical appliances, and construction due to their excellent chemical resistance, low surface energy, good thermal stability, and dielectric properties, they cannot be processed using the phase change or melt processing methods due to their resistance to most solvents and high melt viscosity. Therefore, most traditional PTFE porous membranes or hollow fiber membranes are formed through mechanical methods, including stretching and paste extrusion. However, the above methods are relatively complex, consume a large amount of energy, and pollute the environment. Fortunately, PTFE nanofiber membrane can be prepared by electrospinning; for example, different amounts of PVA or polyethylene oxide (PEO) can be mixed into the PTFE lotion, which can be easily electrospun into fibers and the membrane and then sintered to obtain the porous PTFE membrane.

### 2.6. Reinforcement Materials

Due to their ultra-fine size, high molecular chain orientation, high crystallinity, high strength, and ultra-high specific surface area, electrospun polymer nanofibers can be used as the reinforcing fillers to improve the strength and modulus of the polymer matrix [52]. The preparation method of such nanocomposites mainly includes layer-by-layer coating, immersion coating, impregnation, and casting. In order to achieve a good reinforcement effect, it is necessary to have a good interface between the nanofibers and the polymer matrix, that is, close contact and firm bonding. Therefore, the polymer matrix needs to have a good impregnation effect on the nanofiber fillers.

Gavande et al. prepared a highly transparent and flexible nanocomposite using PK nanofibers to enhance the UV-curable polyurethane acrylate (PUA) [53]. The specific process was electrospinning using dichloromethane and trifluoroacetic acid as solvents. They found that a very small amount of PK nanofibers could increase the yield strength and Young’s modulus of the composite film by 61% and 60%, respectively, increasing its maximum pencil hardness from 2B to HB. They also developed UV-cured PUA-based transparent nanocomposites using electrospun PA6 nanofibers as reinforcing materials [54]. It was found that the addition of PA6 nanofibers could increase the tensile strength of PUA nanocomposite membranes by 85% while basically not affecting the transparency and flexibility of the PUA matrix. It was mainly due to the formation of a large number of hydrogen bonds between the filler and the matrix, which led to good interfacial performance between the two so that the stress could be fully transferred from the PUA matrix to the PA6 nanofibers.

In order to predict the tensile strength of electrospun PA 6 nanofiber-reinforced PUA composites, Gavande et al. used traditional Pukanszky, Nicolais–Narkis, Halpin–Tsai, and Neilson models, while for Young’s modulus, Halpin–Chai, modified Halpin–Tsai, and Hui–Shia models were used [55]. It was found that the theoretical results predicted using the Pukanszky model and the Nicolais–Narkis model were very consistent with the experimental values of the tensile strength of the nanofiber-reinforced nanocomposites. Meanwhile, the Neilson and Halpin–Tsai tensile strength models showed higher binding values than the experimental values with higher volume fractions of nanofibers in the composites. The theoretical values predicted using the modified Halpin–Tsai model were very consistent with the available experimental values of Young’s modulus, while Young’s modulus values evaluated using the Hui–Shia model and the Halpin–Tsai model were the least consistent with the experimental values.

The brittle fracture of epoxy resin materials is an important drawback in their performance, and Daelemans et al. found that adding electrospun thermoplastic nanofibers was a feasible strategy to improve their toughness [56]. They added 6 wt% PA69 and PCL nanofibers to the epoxy resin and found that their fracture energy was increased by 50–100%. In addition, it exhibited a longer stable crack length and required more energy to propagate cracks. They also explored its toughening mechanism. Gavande et al. prepared electrospun PA6 nanofiber-reinforced epoxy matrix composites using vacuum infiltration technology [57]. It was found that the reinforcement of epoxy resin matrix by PA6 nanofibers not only significantly improved the mechanical properties of composites but also significantly enhanced their thermal stability.

Zhang et al. prepared the TPU/SiO_2_ composite nanofibers by electrospinning using colloidal SiO_2_ as the raw material and dimethylformamide (DMF) as the solvent [58]. It was found that when the SiO_2_ content was below 10 wt%, the average diameter of the nanofiber was about 800 nm, and the SiO_2_ nanoparticles had a fairly uniform distribution in the PU nanofibers, not just enriched on the fiber surface. In addition, the addition of SiO_2_ nanoparticles did not reduce the fiber’s ductility but significantly increased the mechanical strength, such as tensile modulus and storage modulus. After further treatment with 3-aminopropyltriethoxysilane, the composite nanofiber felt could adsorb gold nanoparticles and be further used as functional fibers.

Tang et al. electrospun the partially miscible cellulose acetate and PU in a mixed solvent of *N*,*N*-dimethylacetamide (DMAc)/acetone (2/1, *vol*/*vol*) to prepare the continuous cellulose acetate/PU composite nanofibers [59]. It was found that the two components had a co-continuous microstructure, while the PU component improved the tensile strength of the nanofiber felt, and the cellulose acetate component enhanced the rigidity and dimensional stability of the nanofiber felt. The performance of the composite fiber was far superior to that of a single component.

## 3. Discussion and Outlook

Table 1 lists some typical materials, designed structures, key properties, and potential applications of electrospun nanofibers overviewed in this review. It can be seen that although there has been significant progress in the preparation technology and practical application of electrospun polymer composite nanofibers, there are still considerable shortcomings, and the future development directions may focus on the following aspects:

(1) n terms of the product cost of PU-based nanofibers, although PU belongs to a cheaper category compared to other polymer materials, its preparation process is still relatively expensive because it requires the use of a large amount of organic solvents, e.g., DMAc and DMF, which are usually not cheap. Moreover, due to the relatively low production efficiency of electrospinning compared to other preparation processes, the equipment occupation time and energy consumption are usually costly. All of these factors contribute to the relatively high price of PU-based nanofibers compared to other polymer products. Therefore, further development of electrospinning technology is still needed, such as upgrading needleless and multi-needle electrospinning technology and multi-material co-spinning.
(2) The typically large amount of usually corrosive and toxic organic solvents for the electrospinning polymer solutions are prone to environmental pollution. Therefore, much more effort should also be made to develop environmentally friendly solvents, such as water-soluble solvents and recyclable solvents. The combination of the electrospinning method and other related technologies will also be one of the strategies for achieving significant breakthroughs in preparation efficiency. Moreover, water-soluble polymers and eco-friendly melt electrospinning are also excellent choices. According to statistics, the total amount of PU waste accounts for about 30% of the total volume of all waste plastics, which has caused serious environmental problems. Fortunately, most PU materials have both hydrolytic and biodegradable abilities, mainly due to their unique chemical structure. Among them, polyester-type PU has better biodegradability than polyether-type PU. Intentionally selecting the chemical structure can significantly increase PU’s hydrolysis rate. Of course, both hydrolysis and biodegradation require huge storage spaces and take a long time. Therefore, how to further improve the hydrolysis and biodegradation ability of PU remains an important research direction.
(3) The morphology and structure of electrospun nanofibers will still be a focus of attention in terms of product regulation. For example, the crimped morphology and fine structures, such as core–shell and hollow structures of nanofibers, have a significant impact on the performance and function of their membranes. Multi-material co-spinning provides large space for the fine design of multi-layer structures and regulation of multi-phase structures, making it an important research and development direction worthy of attention.
(4) The mechanical properties of electrospun nanofibers and their thin membranes still have some obvious shortcomings. Therefore, how to improve the mechanical properties of single nanofibers by improving their continuity, orientation, crystallinity, etc., and how to increase the interaction force between single fibers to improve the mechanical properties of fiber membranes will still be a focus of research and development. The anti-bacterial, hydrophobic, biocompatible, and biodegradable properties of electrospun nanofibers are the basis of their functional performance, and further improvement and more precise regulation are definitely needed. Among them, the dispersion problem of inorganic, metal, and organic nanofillers in the polymer nanofiber matrix is still one of the technical difficulties and needs to be better solved.
(5) Concerning the disadvantages of PU, such as easy aging (susceptible to UV radiation, oxygen, temperature changes, and other factors) and low mechanical strength and modulus, not only physical treatment but also chemical modification is needed to improve its comprehensive properties. The combination with nanotechnology, e.g., adding nano-size fillers, is one of the most feasible strategies to overcome the disadvantages of PU as the matrix of nanofiber composites.

## 4. Conclusions

As one of the most promising nanomaterials in the 21st century, nanofibers have become a research and development hotspot due to their numerous excellent properties, and electrospinning is the main preparation method for nanofibers and their thin membranes. Combining electrospinning with nanocomposite technology can further improve the mechanical strength and multi-functionality of nanofibers and their thin membranes. This review, represented by the PU-based composite nanofibers, has investigated their latest practical applications in many fields, including sound-absorbing materials, biomedical materials, wearable sensing devices and energy-harvesting devices, adsorbent materials, electromagnetic shielding materials, and reinforcement materials.

The main conclusions are drawn as follows:

(1) In response to noise pollution, especially low-frequency noises below 500 Hz and intermediate-frequency noises of 500–2500 Hz, nanofiber membranes have excellent sound absorption ability due to their involvement in both porous and resonant mechanisms. Combining nanofiber membranes with traditional microfibers can solve the problem of poor sound absorption performance in the low frequency range, achieving the best comprehensive sound absorption effect. Moreover, the addition of nanofiber membranes will barely increase the materials’ thickness and weight.
(2) The application of electrospun nanofibers in the biomedical field mainly includes implantable materials (such as scaffolds) in tissue engineering, controlled drug release, wound dressings, and health materials. The anisotropic structure of both bone tissue and blood vessels can be achieved by utilizing the highly oriented properties of electrospun nanofibers. The polymer nanofibers prepared by electrospinning have more advantages than other nanoparticles when used in targeted drug delivery systems. The bioactive wound dressing prepared by the electrospinning nanofiber membrane has good anti-bacterial properties and can help with wound healing. The electrospinning nanofibers combined with functional materials such as tourmaline can also be used for health materials such as negative ions. For the biomedical applications of electrospun nanofiber membranes, such as wound dressings, traditional materials such as gauze and nonwoven fabrics are used as benchmarks. Although the preparation efficiency of electrospun nanofiber membranes is lower and the price per unit volume and weight is higher, they have some special properties that other materials do not have, so they still have their application market. Of course, combining electrospun nanofiber membranes with traditional materials may achieve synergistic effects in terms of functionality and economy.
(3) In terms of wearable sensing devices, the conductive electrospun nanofibers using nanocomposite technology are suitable for the key components of flexible electronic devices due to their excellent comprehensive performance and can be used to detect the health status of the body and the movement status of different human parts. In terms of energy-harvesting devices, electrospun nanofibers can be used to prepare nanogenerators based on electrostatic induction and contact electrification mechanisms, providing electricity for wearable devices.
(4) In terms of the adsorbent materials, the treatment of oil pollution caused by offshore oil exploration and transportation accidents requires cost-effective oil adsorbents, such as magnetic electrospun composite nanofiber membranes.
(5) In terms of electromagnetic shielding materials, reducing electromagnetic radiation pollution requires the development of high-performance electromagnetic shielding materials, such as conductive polymer composites, which replace traditional metal materials due to their numerous advantages. The conductive electrospun composite nanofiber membrane is one of the outstanding types.
(6) In terms of the reinforcing materials, the high modulus, strength, and ultra-high specific surface area of electrospun polymer nanofibers make them suitable as nanofillers for polymer composites. Their high specific surface area is especially conducive to the impregnation of fillers by the matrix, thus forming a good interface with tight contact and strong bonding. The biggest difference between this review and other reviews related to electrospinning is the emphasis on the combination of composite material technology and electrospinning technology. The introduction of nanofillers can not only improve the mechanical strength of electrospun nanofibers but also endow the material with more functions. This is undoubtedly extremely important for its practical application.
(7) There is still a significant gap in the preparation efficiency, production scale, and product price of electrospun nanofibers compared to the microfibers prepared by traditional methods. Therefore, much more effort in material synthesis, structure design, technique improvement, and so on is still needed for the wider applications of such novel materials.

Although this review has covered most of the important applications of electrospun nanofibers, there are still some potentially important future applications that have not been addressed. To the best of our knowledge, with the further improvement of the performance of electrospun nanofiber membranes, they can be used as electrolyte filtration membranes, surface soft feel materials, surface ultra-black materials, etc.

## Figures and Tables

**Table 1:** Typical materials, designed structures, key properties, and potential applications of electrospun nanofibers overviewed in this review.

Sequence Number	Materials	Designed Structures	Key Properties	Potential Applications	Reference Number
1	PS nanofiber, wool substrate	Self-crimped micro-nanofiber	Improved flexibility, elasticity, and spatial scalability	Water conduction, heat preservation, and functional carrier	[7]
2	PU nanofiber, PET nonwoven	Nanofiber web-reinforced nonwoven and sandwich	High noise reduction coefficient	Sound absorption	[13]
3	PVB nanofiber, PET fiber, TPU film	Bi-layer or three-layer laminate	High sound absorption rate	Sound absorption	[16]
4	PAN nanofiber, PVAc nanofiber, PP nonwoven	Core-shell or hollow nanofibrous membrane	Improved acoustic performance	Sound absorption	[17]
5	PCL nanofiber	Tube with outer and inner cell layers	Good elasticity, burst resistance, and suture retention	Bionic small-diameter vascular vessel	[23]
6	TPU nanofiber	Nanofiber grafted by PEG and anchored by silver nanoparticles	Good anti-bacterial stability, low toxicity, and excellent hemocompatibility	Anti-bacterial use	[31]
7	PU nanofiber	Nanofiber added with TN	Improved mechanical strength, high superhydrophilicity, and high bactericidal effect	Superhydrophilic and anti-bacterial use	[34]
8	PU nanofiber	Nanofiber added with tourmaline and GO nanoparticles	Enhanced mechanical properties and negative ions release	Negative ions textiles and wearable energy therapy	[36]
9	TPU nanofiber	Nanofiber decorated with CNT and modified with PDMS	High stretchability and superhydrophobicity	Superhydrophobic strain sensor	[40]
10	TPU nanofiber	Nanofiber added with CNT	High sensitivity	Smart sports bandage	[42]
11	TPU nanofiber	Nanofiber added with GO	High anti-fouling, dye removal, and anti-bacterial properties	Adsorption of organic dyes	[48]
12	PK nanofiber, PUA film	Sandwich	UV curability, high transparency, and high flexibility	Coating for metals and optoelectronic devices	[53]
13	PA 6 nanofiber, PUA film	Bilayer	UV curability and high transparency	Protective coating	[54]

## Abbreviations

1D	one-dimensional
3D	three-dimensional
AC	alternating current
CNT	carbon nanotube
DC	direct current
DMAc	*N*,*N*-dimethylacetamide
DMF	dimethylformamide
GO	graphene oxide
MOF	metal–organic framework
MWCNT	multi-wall carbon nanotube
MXene	two-dimensional transition metal carbon nitrides
PA	polyamide
PAN	polyacrylonitrile
PCL	polycaprolactone
PDA	polydopamine
PDLA	poly(*D*-lactic acid)
PDMS	polydimethyl siloxane
PEG	polyethylene glycol
PEO	polyethylene oxide
PFDT	1H,1H,2H,2H-perfluorododecanethiol
PET	polyethylene terephthalate
PK	polyketone
PLA	polylactic acid
PLLA	poly(*L*-lactic acid)
PMMA	polymethyl methacrylate
PP	polypropylene
PS	polystyrene
PTFE	polytetrafluoroethylene
PU	polyurethane
PUA	polyurethane acrylate
PVA	polyvinyl alcohol
PVAc	polyvinyl acetate
PVB	polyvinyl butyral
PVDF	polyvinylidene fluoride
PZT	lead zirconate titanate
SEM	scanning electron microscope
TENG	triboelectric nanogenerator
TM	tourmaline
TN	tourmaline nanoparticles
TPU	thermoplastic polyurethane
UV	ultraviolet
